# Development and validation of the angiotensin-converting enzyme inhibitor (ACEI) induced angioedema investigator rating scale and proposed discharge criteria

**DOI:** 10.1186/s12913-017-2274-4

**Published:** 2017-05-22

**Authors:** Nicola Bonner, Charlotte Panter, Alan Kimura, Rich Sinert, Joseph Moellman, Jonathan A. Bernstein

**Affiliations:** 1 Adelphi Values, Adelphi Mill, Grimshaw Lane, Bollington, Cheshire SK10 5JB UK; 2 Shire, 300 Shire Way, Lexington, MA 02421 USA; 3 Department of Emergency Medicine, SUNY Downstate Medical Center and Kings County Hospital Center, Brooklyn, NY USA; 4 Department of Emergency Medicine, University of Cincinnati College of Medicine, Cincinnati, OH USA; 5 Clinical Research, Department of Internal Medicine, Division of Immunology/Allergy Section, University of Cincinnati College of Medicine, Cincinnati, OH USA

**Keywords:** Angiotensin-converting enzyme inhibitors (ACEI), ACEI-Angioedema (ACEI-A), Severity scoring, Discharge criteria, Rating scale

## Abstract

**Background:**

The use of angiotensin-converting enzyme inhibitors (ACEI) has been associated with the development of bradykinin-mediated angioedema. With ever-widening indications for ACEI in diseases including hypertension, congestive heart failure and diabetic nephropathy, a concomitant increase in ACEI-Angioedema (ACEI-A) has been reported. At present there is no validated severity scoring or discharge criteria for ACEI-A. We sought to develop and validate an investigator rating scale with corresponding discharge criteria using clinicians experienced in treating ACEI-A.

**Methods:**

In-depth, 60-min qualitative telephone interviews were conducted with 12 US-based emergency physicians. Beforehand, clinicians were sent four case studies describing patients experiencing different severities of angioedema attacks. Clinicians were initially asked open-ended questions about their experience of patients’ symptoms, treatment and discharge decisions. Clinicians then rated each patient case study and discussed patient diagnoses, ratings of symptom severity and discharge evaluation. The ratings were used to assess inter-rater reliability of the scale using the intra-class correlation coefficient (ICC) using IBM SPSS analysis Version 19 software.

**Results:**

The findings provide support focusing on four key symptoms of airway compromise scored on a 0–4 scale: 1) Difficulty Breathing, 2) Difficulty Swallowing, 3) Voice Changes and 4) Tongue Swelling and the corresponding discharge criteria of a score of 0 or ‘No symptoms’ for Difficulty Breathing and Difficulty Swallowing and a score of 0 or 1 indicating mild or absence of symptoms for Voice Change and Tongue Swelling. Eleven clinicians agreed the absence of standardized discharge criteria supported the use of this scale. All physicians concurred with the recommended discharge criteria. The clinician ratings provided evidence of strong inter-rater reliability for the rating scale (ICC > 0.80).

**Conclusion:**

The investigator rating scale and discharge criteria are clinically valid, relevant and reliable. Moreover, both address the current unmet need for standardized ED discharge criteria.

## Background

### Angiotensin-converting enzyme inhibitor induced angioedema

The use of angiotensin-converting enzyme inhibitors (ACEI) has been associated with the development of bradykinin-mediated angioedema.With ever-widening indications for ACEI for diseases including hypertension, congestive heart failure and diabetic nephropathy, a concomitant increase in ACEI-Angioedema (ACEI-A) has been reported [1, 2]. ACEI-A is considered to be potentially life-threatening [2] and although the reported incidence of ACEI-A is low, estimated to occur in 0.1 to 1% of patient’s prescribed with ACEI [3], evidence indicates that approximately 30 to 73% of angioedema cases recorded in Emergency Departments (EDs) are caused by ACEIs [3, 4]. The rate of ACEI-A associated visits to EDs annually is estimated at 0.7 per 100,000 [3] and notably, a higher incidence has been reported in black patients, female patients, smokers, those aged 65 years, and those with a history of cough associated with ACEI use [5].

ACEI-A is characterized by a localized, non-pitting, non-pruritic, asymmetric swelling of sub-epithelial and sub-mucosal tissues which typically involves surfaces that have loose connective tissue such as the head, neck, lips, mouth, tongue, pharynx, and larynx.[6, 7] Tongue swelling may be notably prominent and is a significant predictor that a patient may require hospitalization and possible airway intervention [8, 9]. Involvement of the upper respiratory tract may progress to serious acute respiratory distress, airway obstruction, and death due to asphyxiation [10].

ACEI-A may present at any time during ACEI therapy and tends to be more severe and longer lasting than histamine-mediated angioedema [11]. Most cases occur during the first week of ACEI treatment and up to 25% of cases occur within the first month of treatment [12]. However, delayed onset is not unusual, where patients may develop ACEI-A after years of treatment [13]. From onset the angioedema may worsen within minutes to hours, and the overall time course may vary depending on the severity of the episode, ranging from several hours to several days [6, 14].

Angioedema resulting from ACEIs is considered to be mediated by excessive levels of bradykinin, that previously have been most notably associated with hereditary conditions [11]. Bradykinin, a potent vasodilator increases vascular permeability and leads to rapid accumulation of fluid in the interstitial tissues. ACE inhibitors directly block the activity of ACE, the main bradykinin-inactivating peptidase in humans, resulting in increased systemic levels of bradykinin. Nussberger and colleagues have demonstrated elevated plasma concentrations of bradykinin in patients on ACE inhibitors, as well as in a patient with ACEI-A [15, 16].

To date, there is no approved definitive treatment for ACEI-A. When managing suspected cases, the most important step is to permanently discontinue ACEI therapy and to provide the appropriate supportive care; the majority of patients require simple observation only. Patients with life-threatening edema of the upper airway may need to undergo an invasive procedure such as intubation, tracheotomy, or cricothyroidotomy [17, 18]. Prolonged hospital stays, possibly in the intensive care unit, may also occur due to the slow resolution of ACEI-A symptoms [14, 18].

### Development of treatments for ACEI

ACEI-A patients, when presenting to emergency department (ED), do not respond to the conventional therapies (comprised of antihistamines, epinephrine and corticosteroids) as do patients with histamine-mediated angioedema [11]. It has therefore become paramount that physicians are able to distinguish between histamine- and non-histamine mediated angioedema. The latter should be suspected in the absence of pruritus and urticaria, and when angioedema is associated with abdominal symptoms [11]. However, despite the potentially life-threatening nature of the condition, there are currently no approved pharmacological treatments for ACEI-A. An effective pharmacologic therapy for ACEI-A would address a significant unmet medical need; therefore, studies to assess potential treatments have been undertaken.

A recently completed randomized, controlled, phase II trial compared conventional therapy with ecallantide (a recombinant plasma kallikrein inhibitor approved for treatment in HAE) to placebo and conventional therapy respectively in ED patients with ACEI-A [3]. A non-statistical trend to a larger proportion of ACEI-A patients receiving ecallantide met discharge criteria in four hours or less, compared to patients receiving placebo [3].

Additionally, icatibant is a potent, highly selective antagonist of the bradykinin B2 receptor, which may be a useful therapeutic approach for the treatment of ACEI-A [17, 19–21]. A recently completed double-blind, randomized, controlled phase II trial evaluated a 30 mg dose of sub-cutaneously administered icatibant in patients with ACEI-A. Investigators assessed the severity of six symptoms (pain, shortness of breath, dysphagia, change in voice, sensation of a foreign body, and feeling of pressure), using a scale from 0 (no symptoms) to 3 (severe symptoms) [17]. The time to onset of symptom relief was defined as the time to the first improvement (i.e. decrease) of at least one point in the composite score of the investigator-assessed symptom scores [22]. Among patients with ACEI-A, the median time to the onset of symptom relief (according to a composite investigator-assessed symptom score) and time to complete resolution of edema was significantly shorter with icatibant than with standard therapy consisting of a glucocorticoid and an antihistamine [22]. Although this scale was used in the phase II trial, it had not been validated.

### The need for a severity rating scale and standardized discharge criteria

Ishoo et al. presented a staging system to predict airway risk associated with ACEI-A based on anatomic site of symptom presentation [8]. While the Ishoo criteria provides one potential way for a physician to evaluate risk associated with ACEI-A and admission decisions, the criteria require the use of a laryngoscopy to stage a patient and more importantly, the criteria have not yet been validated [11]. At present there are no validated ACEI-A severity scoring systems focusing on the most important airway symptoms or agreed criteria for discharge following treatment. This highlights an unmet need for ED physicians and the importance of development of a standardized validated rating system for the assessment of ACEI-A for use in clinical trials designed to evaluate new treatments as well as in clinical practice.

### Development of severity rating scale and proposed discharge criteria, and specific intended context of use

The efficacy and safety of icatibant is being evaluated in a phase III, randomized, placebo-controlled trial, which has now completed (NCT01919801). The primary endpoint of the study is the time to meeting discharge criteria (TMDC); that is, the time at which a patient can be safely discharged following treatment. However, given the absence of validated measures to assess the severity of airway symptoms associated with ACEI-A, the development of an ACEI-A airway symptom severity rating scale (‘Investigator rating scale’) and accompanying discharge criteria was undertaken. The aim of this instrument development program was to address the gap in available assessments for airway symptoms associated with ACEI-A.

**Fig. 1:**
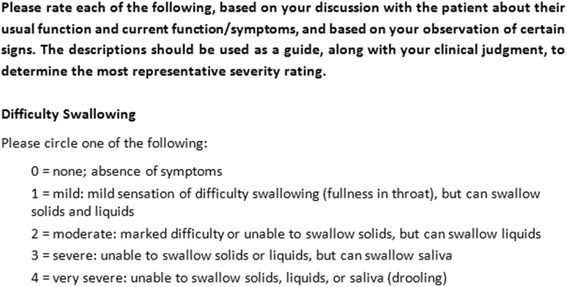
Extract of the investigator rating scale: severity rating scale for difficulty swallowing

A review of published literature to determine key ACE-I symptoms was conducted. This was followed by a meeting involving outcomes development and clinical experts to develop the Investigator rating scale and proposed discharge criteria for use in the phase III clinical trial. The five-point investigator rating scale assesses four symptoms/signs associated with airway compromise caused by ACEI-A: difficulty breathing, difficulty swallowing, voice changes and tongue swelling. These symptoms are consistent with those noted to be at risk for requiring airway intervention by Ishoo et al. [8]. The rating scale has a response scale specific to each symptom, ranging from 0 to 4, with 0 being ‘none; absence of symptoms’ and 4 being ‘very severe’ with specific descriptions relative to each of the four symptoms to guide clinicians (alongside their own clinical judgment) to determine the most representative severity rating (see Fig. 1). Improvement is defined by a reduction in score.

The acceptable discharge criteria following treatment is proposed to be defined as the first time point when complete resolution of any breathing difficulty or difficulty swallowing (investigator score = 0), and resolution of tongue edema and voice change to at least mild in severity (investigator score = 1) has occurred. The investigator scores are based on the criteria of the ‘Investigator Rating Scale’.

### Specific aims and objectives of this particular paper

This study aimed to confirm the clinical relevance, content validity, and reliability of the scale and proposed discharge criteria with clinicians experienced in treating ACEI-A, as well as determine the inter-rater reliability of clinician ratings of discharge criteria. Furthermore, the study aimed to explore the change in the level of symptom severity that would define a clinically meaningful improvement in a patient’s condition.

## Methods

### Overview of study methods

**Fig. 2:**
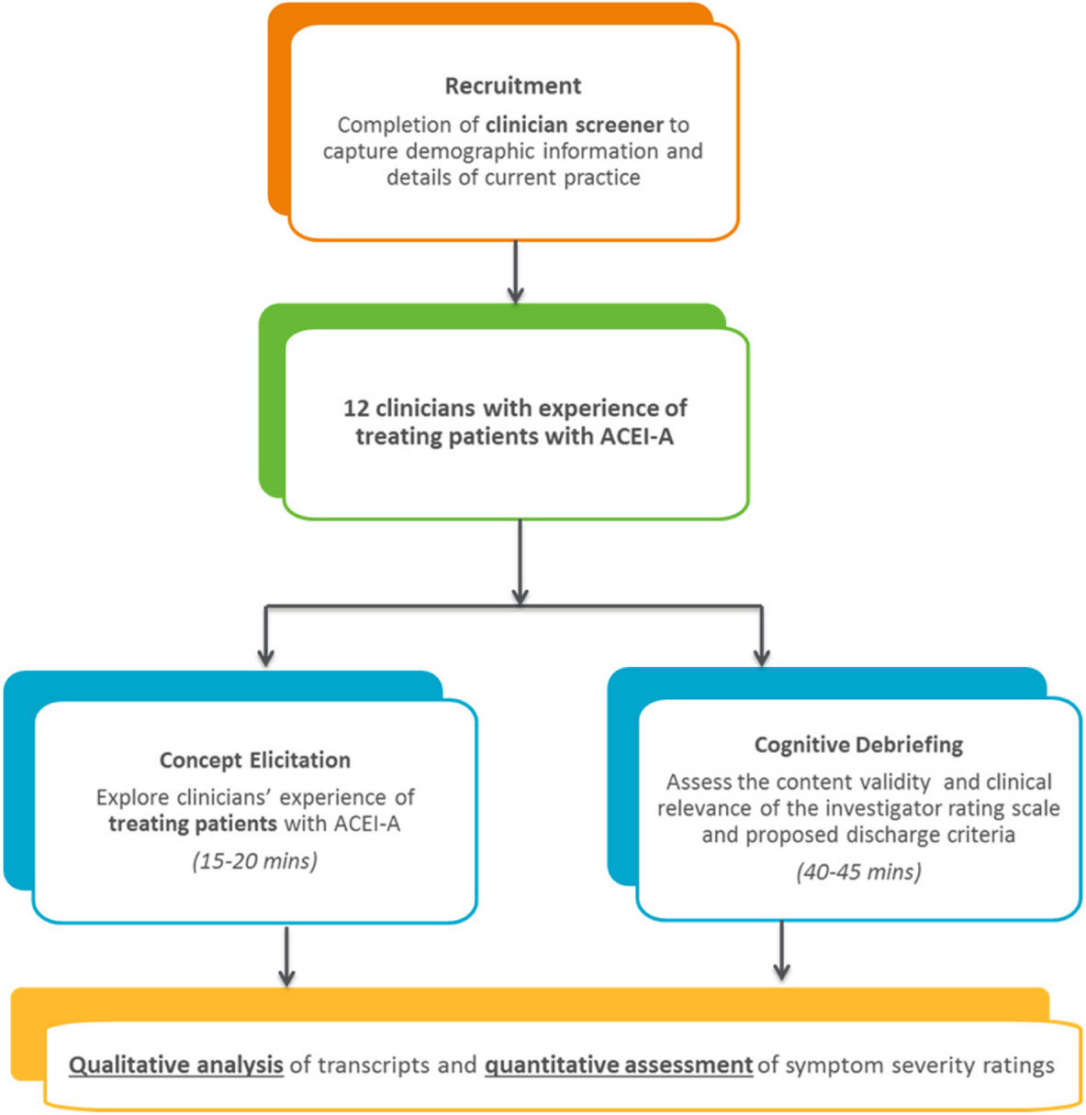
Overview of study methodology

This was a non-interventional, qualitative, interview study involving 12 clinicians in the US who have experience treating patients with ACEI-A. As illustrated in the methods flow diagram (see Fig. 2 below), the interview was structured into two parts: an exploratory concept elicitation section involving an open-ended discussion of the clinician’s experience with patients who have suffered from ACEI-A attacks, and a cognitive debriefing section to evaluate the content validity of the investigator rating scale.

### Recruitment

Clinicians were recruited from a convenience sample of clinicians based at two specialist angioedema centres in the USA (Brooklyn, NY and Cincinnati, Ohio) through two clinical experts involved in the study (RS and JB). RS and JB were initially identified for their involvement in the study by Shire, RS and JB then worked with Adelphi Values to recruit clinicians into the study. All clinicians were attending ED physicians). Clinicians were initially approached by RS or JB to confirm their interest in the study, and their details were then passed on to NB and CP. The aim and process of the study was described to the clinicians by NB and CP via email and telephone, and if they agreed to take part, clinicians were asked to complete a clinician screener to collect demographic and background information about the clinician and the nature of the clinician’s practice with patients. Once clinicians had completed the screener, a mutually agreed time for the interview was arranged and arrangements were made to send the relevant interview materials to the clinicians.

### Ethics, consent and permissions

Given the non-interventional nature of this study and the fact that the study only involved clinicians and no patients, no specific ethical approval for the study was sought. However, all participants provided their written informed consent to take part in the study and all study activities were conducted in accordance with principles outlined in the Declaration of Helsinki.

### Interview materials

Prior to the interview, clinicians were sent four patient case studies and the investigator rating scale by mail. The case studies described patients who were experiencing different types of angioedema attacks (ACEI-A, hereditary angioedema (HAE) and allergic angioedema). Clinicians were asked not to view the interview materials until instructed during the interview to maintain the scientific integrity of the study and to avoid biasing their responses during the interview. At the relevant point in the interview the clinicians were asked to open the envelope containing the interview materials.

### Interview process

Each interview lasted approximately 60 min (15–20 min of concept elicitation and 40–45 min of cognitive debriefing). A detailed interview guide, rather than a script, was developed and used by the trained interviewer. Following the first two clinician interviews the guide was reviewed for any necessary revisions; however, it was agreed by the researchers that no changes were required. The clinicians took part in a single interview. All interviews were conducted via telephone and involved only the interviewer and the clinician taking part in the interview. NB and CP, who are experienced qualitative interviewers and have received training in qualitative interview techniques, conducted all interviews. Clinicians were told at the start of the interview that interviews were being conducted by NB and CP on behalf of Shire.

The concept elicitation section of the interview was conducted first and involved broad open-ended exploratory questions, designed to facilitate spontaneous and non-biased elicitation of content regarding the clinician’s experience with ACEI-A patients. Specifically, this included exploring the symptoms they look for in patients, how they treat patients, and how discharge decisions are typically made from a clinical perspective. More direct, focused questions were used towards the end of the interview if concepts of interest had not been spontaneously elicited or fully explored with study subjects.

**Fig. 3:**
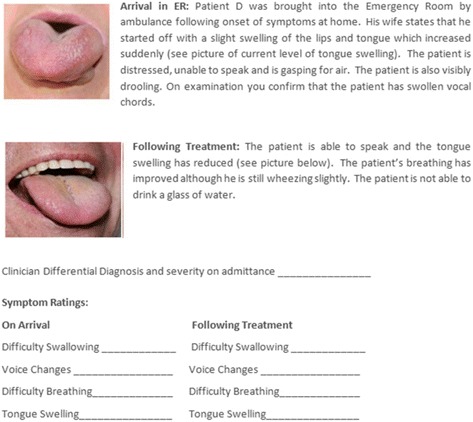
Example of patient case study used in the interview

The cognitive debriefing section of the interview assessed the content validity and clinical relevance of the investigator scale and the proposed discharge criteria. The clinicians were asked to open the envelope containing the relevant interview materials and were asked to read four patient case studies describing different severities of angioedema attacks before and following treatment (Fig. 3). Clinicians provided a diagnosis for the type of angioedema experienced in each case study and a rating for the severity of the four symptoms assessed by the investigator rating scale (difficulty swallowing, voice changes, difficulty breathing and tongue swelling). Symptom severity ratings were obtained for the patient description before and following treatment. Table 1 presents an example of some of the questions asked during the interviews for both the concept elicitation and cognitive debriefing phases.

**Table 1:** Example clinician 1 interview questions

Interview stage	Example interview questions
Concept elicitation: Open ended questions	• “Please tell me about the symptoms that patients experience while suffering from an ACE inhibitor-induced angioedema attack?” • “What symptoms are most frequently reported by patients?” • “Where on the body do symptoms of ACE inhibitor induced angioedema tend to occur?”
Concept elicitation: Direct, focused questions	• “How would you usually treat patients with ACE inhibitor-induced angioedema?” • “How do you decide when a patient is ready to be discharged? What symptoms do you evaluate? Do you weigh some symptoms more than others? How?” • “On a scale from 0–4, with 0 being none; absence of symptoms and 4 being very severe, in your opinion what reduction would constitute a clinically meaningful improvement in each of the symptoms of an ACE inhibitor-induced angioedema attack?” • Using the same scale (0 = none to 4 = very severe), to what level would they need to get to (for each symptom), in order to feel comfortable discharging them? Are some symptoms more important than others?
Cognitive debriefing	• “Please read case study A and talk me through your thoughts and decisions before rating the patient’s symptoms using the scale provided” • “What diagnosis did you give this patient? What led you to this decision?” • “What rating did you give for the symptom Difficulty Swallowing? What led you to this decision?” • “At what point would you consider that this patient could be discharged from hospital? (probe for each symptom) Do you think this patient can be discharged based on the information given about their current state?” • “Do you feel that this [discharge] criteria is clinically meaningful?” • “Do you feel this assessment captures all of the key symptoms of ACE inhibitor-induced angioedema?”

Throughout this section of the interview, clinicians were asked to use a ‘think aloud’ approach [23, 24], where they were asked to share their thoughts and reasoning for selecting each response. Specifically, clinicians were asked to talk through their patient diagnoses and symptom severity ratings, and describe how they came to their response. The interviewer further asked specific probes to obtain insights into the clinician’s interpretation and understanding of each case study. Clinicians also discussed whether they would discharge the patient based on their current symptom experience. Finally clinicians were provided with the proposed discharge criteria and asked whether they considered it an appropriate point to discharge a patient. All interviews were audio-recorded and transcribed verbatim with any identifiable information removed. Participants did not provide any input on the study findings following the interviews.

### Qualitative analysis of interview transcripts

Verbatim transcripts of the interviews were subject to thematic analysis by NB and CP [24–26] using Atlas.Ti software (Version 7, ATLAS.ti Scientific Software Development GmbH, Berlin, Germany). Each transcript was assessed and clinician comments that pertained to our main research questions were highlighted. A coding scheme was then created to be used throughout the analysis process (each highlighted statement was eventually coded). New codes were organically added to the structure as themes emerged from the data and analysis progressed. After analysing each transcript, the coded statements were then moved into their relevant concepts verbatim. Thematic methods were not appropriate to use for analysis of the cognitive debriefing component of interviews. Here, codes were assigned to each case study and then further codes were applied to indicate the patient diagnosis, symptom severity ratings and the point at which the patient would be discharged. Finally, additional codes were applied to indicate whether the proposed discharge criteria were appropriate. Given the relatively small sample size and the fact that cognitive debriefing of the investigator rating scale and the proposed discharge criteria was the key objective of the study assessing evidence of data saturation was not a primary focus of this study.

### Quantitative analysis of clinician ratings

The clinician ratings on each case study were used for the purpose of assessing inter-rater reliability of the investigator rating scale (i.e. the clinicians’ agreement on the ratings of symptoms for each of the patient case studies). To assess overall inter-rater reliability an intra-class correlation (ICC) coefficient was used. According to the system proposed by Shrout and Fleiss [27], this was a case 2 ICC and was assessed using a two-way random-effects analysis of variance (ANOVA) on IBM SPSS Statistics [28]. An ICC coefficient > .70 is generally considered evidence of adequate inter-rater reliability [29, 30].

## Results

### Clinician sample characteristics

Clinicians with a diverse range of experience were targeted; all 12 clinicians who agreed to take part in the study completed the interview; there were no drop outs prior to study participation. The key demographic and clinical experience characteristics of the 12 clinicians are shown in Table 2.

**Table 2:** Clinician sample demographic information and 1 clinical experience

Description	N (%)
Gender	
Male	8 (66.7%)
Female	4 (33.3%)
Age	
Min	33
Max	60
Average	41.42
Clinician Speciality	
Emergency Medicine	11 (91.7%)
Emergency Medicine/Palliative Care	1 (8.3%)
Interest in Angioedema	
Yes	7 (58.3%)
No	5 (41.7%)
Position	
Attending Physician in Emergency Department	12 (100%)
Length of time in role (years)	
Min	3
Max	20
Average	9.58
Setting	
University Hospital	7 (58.3%)
University Hospital and Community or General Hospital	5 (41.7%)
No. of angioedema patients seen in a typical month	
Min	1
Max	14
Experience of measuring severity of ACEI symptoms	
Yes	7 (58.3%)
No	5 (41.7%)
Experience of measuring severity of angioedema symptoms	
Yes	6 (50%)
No	6 (50%)

Most clinicians were male (66.7%) and the average age was 41 years (range: 33–60 years). All clinicians were attending ED physicians with most working solely in a university hospital setting (58.3%); the others worked in both a university and community/general hospital setting (41.7%). The average length of time as an ED physician was 9.5 years post-residency (range: 3–20 years). The clinicians reported seeing between one and 14 angioedema patients in a typical month. The majority of angioedema cases seen by the clinicians were ACEI-A attacks; specifically, 0–10% of cases seen by clinicians were acquired angioedema, 0–25% of cases seen by the clinicians were HAE, 0–30% of cases seen by the clinicians were allergic angioedema and 0–95% of cases seen by the clinicians were cases of ACEI-A. It is worth noting that clinicians were recruited from two specialist angioedema centres in the US so the number of cases seen by these clinicians is likely skewed compared to the general population of the US. Most of the clinicians (58.3%, *n* = 7) reported having experience in measuring the severity of ACEI-A symptoms, while 50% (*n* = 6) of the clinicians reported having experience in measuring the severity of angioedema symptoms (regardless of etiology or specific location). Of those clinicians, two specified assessing symptoms for admission and airway management during practice, whilst one reported gaining experience as part of a number of clinical trials involving ACEI-A and HAE patients.

Most clinicians (58.3%) had a particular interest in the area of angioedema, with some further specifying the following: clinical care of ACEI-A patients, research and treatment, airway management and/or therapies to avoid the use of airway management. One clinician (JM) reported an invested interest in ACEI-A having been involved in a study for a clinical drug trial, published multiple articles on angioedema and named co-editor of a consensus statement for the emergency management of angioedema [11].

### Concept elicitation findings: clinicians’ experiences of seeing ACEI-A patients

#### Symptoms of ACEI-A

**Fig. 4:**
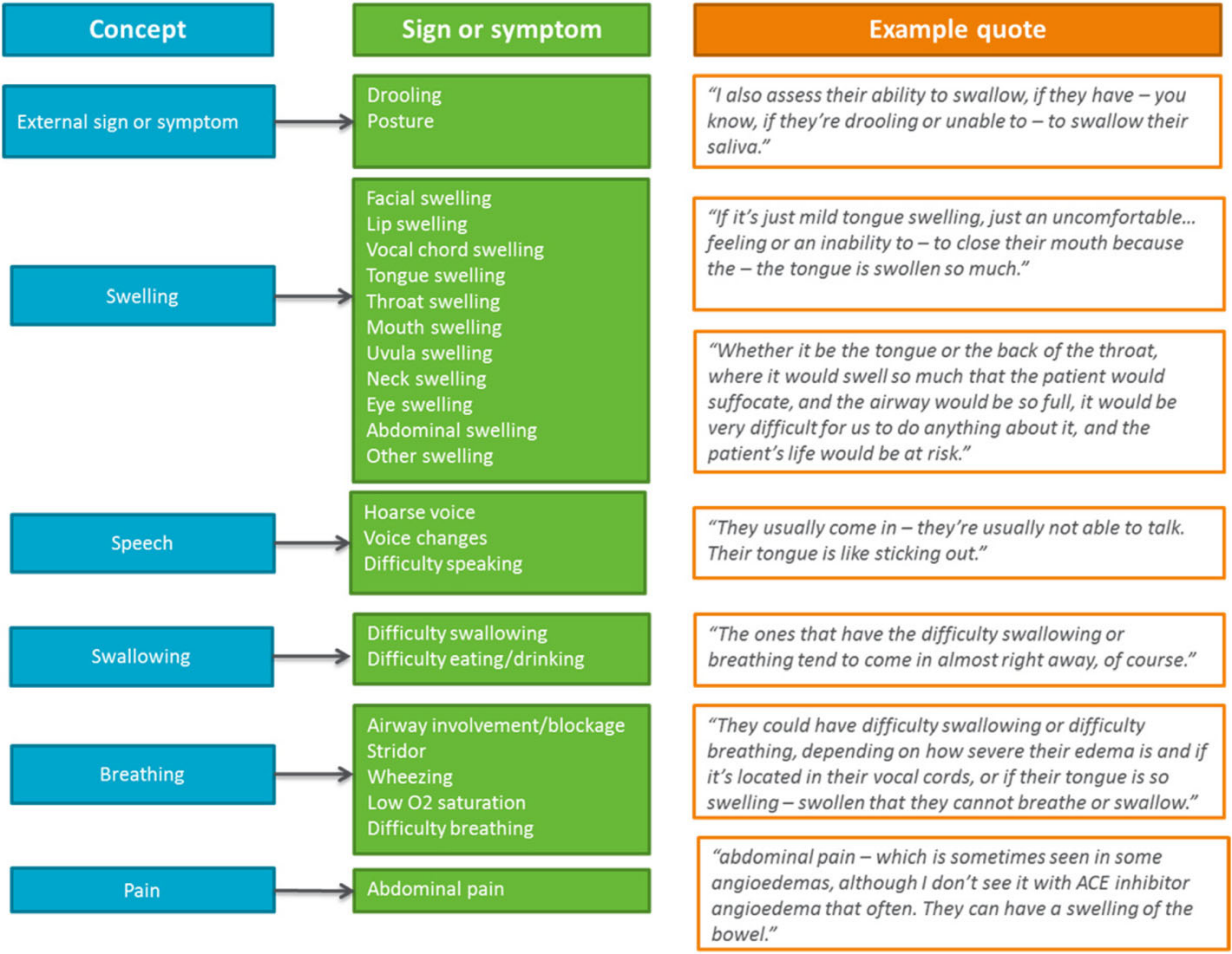
Conceptual model of ACEI-A signs and symptoms as reported by clinicians

Figure 4 presents the signs and symptoms of ACEI-A as reported by clinicians by concept, with example quotes. The most common symptoms and signs reported by clinicians included difficulty breathing (*n* = 12), lip swelling (*n* = 12), vocal cord swelling (*n* = 11), tongue swelling (*n* = 11), facial swelling (*n* = 10), voice changes (*n* = 10) and difficulty swallowing (*n* = 9).

Clinicians tended to talk about a collection of symptoms occurring in patients with ACEI-A. All clinicians mentioned lip swelling amongst the most frequently reported symptoms, followed by tongue swelling (*n* = 8). For example, *“Either my lips are swelling or my – my tongue feels like it’s swelling. That’s probably 98% of how they present”.* Difficulty breathing was reported to be the most concerning symptom to patients (*n* = 10), as well as to most clinicians (*n* = 8).

#### A clinically meaningful improvement in symptoms

There was limited consistency across all clinicians as to what would constitute a clinically meaningful improvement in a patient’s condition following treatment. Some clinicians referred to any improvement in certain symptoms being clinically meaningful. Others referred to resolution of specific symptoms (throat swelling (*n* = 4), difficulty breathing (*n* = 3), voice change (*n* = 3) and difficulty swallowing (*n* = 2)).

#### Discharge

To discharge patients, clinicians said they would like complete resolution of difficulty breathing (*n* = 11), difficulty swallowing (*n* = 9) and voice changes (*n* = 8). Most clinicians (*n* = 8) considered discharge acceptable if any tongue swelling reaches mild or moderate on a severity scale. Four clinicians highlighted the importance of patient input and said they would base their decision to discharge the patient on both the evaluation of signs and symptoms via physical exam, and the patient’s subjective experience of the symptoms. For example, *“if I look at the patient and I say… I think your upper lip is still the same size, so it’s still edematous, but if they say, yeah, but it’s less numb and the tingling feels a lot better – the edema’s resolving… I’ll feel more comfortable letting those patients go home”.*

#### Standardized discharge criteria

Eleven clinicians confirmed the lack of standardized criteria currently in practice for discharge of ACEI-A patients and eight of those clinicians confirmed that such criteria would be beneficial.

### Cognitive debriefing findings: content validity and clinical relevance of the investigator rating scale and proposed discharge criteria

#### Investigator rating scale

All clinicians interviewed felt the investigator rating scale captured the key symptoms of ACEI-A that are of clinical concern and relevant when making patient discharge decisions. Further, the investigator rating scale was considered an appropriate measure by the majority of clinicians (*n* = 8); one clinician was unsure of how appropriate the measure would be with regard to capturing change in score, the remaining clinicians did not specifically discuss this question. Four clinicians suggested the addition of several symptoms to the investigator rating scale, which included vocal cord swelling (*n* = 3), uvula swelling (*n* = 1), or lip swelling and vital signs (*n* = 1).

#### Proposed discharge criteria

All clinicians considered the proposed discharge criteria clinically appropriate and agreed there should be complete resolution of difficulty swallowing and difficulty breathing before discharge. Most clinicians agreed with resolution of tongue edema (*n* = 11) to at least mild in severity. Although earlier in the interview (concept elicitation section) eight clinicians suggested complete resolution of voice change would be appropriate for discharge, when asked directly nine agreed with resolution of voice changes to at least mild in severity as appropriate discharge criteria. The remaining three clinicians stated that they would have reservations about voice changes and would like complete resolution.

### Inter-rater reliability of the investigator rating scale

All of the clinicians (*n* = 12) provided severity ratings on the four symptoms (before and following treatment) for each of the four patient case studies. There were no missing data. Of note, where clinicians selected more than one rating for a symptom, the lower score was selected.

The ICC was calculated separately for each symptom rating before treatment and following treatment. Using the investigator rating scale, the symptom ratings on arrival demonstrated excellent inter-rater reliability with an ICC of 0.87. Similarly, the ratings following treatment indicated good inter-rater reliability across clinicians with an ICC of 0.80.

## Discussion

The development of the investigator rating scale and accompanying discharge criteria was driven by the current absence of a standardized validated rating system for the assessment of ACEI-A attacks. Moreover, the rating scale and proposed discharge criteria were required for use in the completed phase III trial (NCT01919801) to evaluate the safety and efficacy of icatibant where TMDC is being assessed as a primary endpoint. However, it is important to note that the rating scale and discharge criteria will also be of substantial benefit to ED physicians when rating the severity of an ACEI-A attack and making treatment decisions and discharge decisions following treatment. This study aimed to confirm the clinical relevance, content validity, and reliability of the scale and proposed discharge criteria with clinicians experienced in treating ACEI-A.

The concept elicitation findings provide support for focus on the four key symptoms of airway compromise in the investigator rating scale; difficulty breathing, difficulty swallowing, voice changes and tongue swelling were amongst the most commonly reported symptoms by clinicians, supporting their importance and relevance. Moreover, all of the other reported symptoms or signs of ACEI-A are each assessed by the investigator rating scale as part of the detailed response options specific to each symptom. Although lip swelling was discussed by the entire sample of clinicians and described as the most frequently reported symptom by patients, lip swelling and other exceptions, such as eye and facial swelling, are considered more ‘cosmetically’ disturbing and unlikely to compromise airway, therefore were not included in the scale. While additional symptoms were suggested for the investigator rating scale, each of the suggested symptoms was captured as part of the detailed response options. Clinicians suggested adding vocal cord swelling, which is captured as part of the voice changes response scale, e.g. *“severe: very difficult to hear speech or for patient to articulate”*. Additionally, clinicians suggested adding uvula swelling; while uvula swelling is used as a landmark to evaluate tongue swelling, uvula swelling would be captured in the scale under difficulty breathing and difficulty swallowing.

The symptom severity ratings collected in the study provided evidence of strong inter-rater reliability of the investigator rating scale between clinicians. Reliability was highest when the clinicians were rating the patient case studies on arrival and slightly lower when rating the patient case studies following treatment, which may be due to the little information that was provided in the post-treatment descriptions for the patient case studies, making it difficult for clinicians to rate symptoms. It is important to highlight that, although clinicians stated it was difficult to rate symptoms with the little information provided, the rating scale still yielded strong inter-rater reliability results for post-treatment, which is extremely encouraging. Nonetheless, this data was collected in a controlled manner, with pre-defined clinical descriptions making it difficult to generalize for use in general clinical practice. Assessment of inter-rater reliability in a larger study, based on actual patient ratings could confirm these results.

The absence of standardized discharge criteria was confirmed and supported the use of the scale and proposed discharge criteria. The proposed discharge criteria were considered to be an appropriate point at which patients could be discharged following treatment, although any discharge decision should be made based on clinician rating and discussion with the patient. As detailed in the instructions of the investigator rating scale, the scale is intended to be used for symptom ratings based on the clinician’s discussion with the patient about their usual function and current function/symptoms, and based on their observation of certain clinical signs. Thus, the overall picture of the patient’s condition can be assessed through use of the rating scale and discussion with the patient.

While there are currently limited criteria or guidelines to direct disposition decisions for hospitalization versus discharge home for ACEI-A patients, the Ishoo criteria provide a working framework for ED physicians in terms of the evaluation and management of bradykinin-mediated angioedema disorders such as ACEI-A [8]. According to the Ishoo criteria, patient disposition for hospital admission or discharge home should be defined according to severity of airway involvement. The clinical characteristics for each stage are as follows: Stage I includes facial rash, facial edema and lip edema; Stage II includes soft palate edema; Stage III includes lingual edema; and Stage IV includes laryngeal edema. The recommended disposition for Stage I-II patients is to be managed as outpatients or admitted to an observation unit (for extended monitoring), and for Stage III-IV patients, it is recommended that select stage III and IV patients are admitted to the intensive care unit as appropriate as these patients are likely to be suffering from respiratory distress or in need or airway intervention.

While the Ishoo criteria provides one potential way for a physician to evaluate risk and admission decisions, the criteria require the use of a laryngoscopy to stage a patient and more importantly, the criteria have not been validated [11]. The results presented from this study support the investigator rating scale as a reliable instrument for assessing ACEI-A which is clinically relevant to ED physicians and is simple to complete. Additionally, use in the completed phase III clinical trial (NCT01919801) will add further evidence to support these characteristics of an instrument addressing an unmet need in diagnosis and assessment of ACEI-A.

## Conclusions

In summary, the findings from the clinician qualitative interviews provided evidence to support the investigator rating scale and proposed discharge criteria as comprehensive, clinically valid, relevant and reliable. Both will have benefits and use in clinical practice in helping physicians rate the severity of an attack to aid treatment decisions and determine the appropriate point at which a patient could be discharged following treatment for an ACEI-A attack. Moreover, both address the current unmet need for standardized discharge criteria.

## Abbreviations

ACEI: Angiotensin-converting enzyme inhibitors
ACEI-A: Angiotensin-converting enzyme inhibitors-Angioedema
ANOVA: Analysis of variance
ED: Emergency Department
ICC: Intra-class correlation coefficient
TMDC: Time to meeting discharge criteria

## References

[1] Lerch M (2012). Drug-induced angioedema. Chem Immunol Allergy.

[2] Cicardi M, Zingale LC, Bergamaschini L, Agostoni A (2004). Angioedema associated with angiotensin-converting enzyme inhibitor use: outcome after switching to a different treatment. Arch Intern Med.

[3] Bernstein JA, Moellman JJ, Collins SP, Hart KW, Lindsell CJ (2015). Effectiveness of ecallantide in treating angiotensin-converting enzyme inhibitor-induced angioedema in the emergency department. Ann Allergy Asthma Immunol.

[4] Chan NJ, Soliman AMS (2015). Angiotensin Converting Enzyme Inhibitor-Related Angioedema: Onset, Presentation, and Management. Ann. Otol. Rhinol. Laryngol.

[5] Lewis LM, Graffeo C, Crosley P, Klausner HA, Clark CL, Frank A (2015). Ecallantide for the Acute Treatment of Angiotensin-Converting Enzyme Inhibitor–Induced Angioedema: A Multicenter, Randomized, Controlled Trial. Ann Emerg Med.

[6] Sica DA, Black HR (2002). Angioedema in heart failure: occurrence with ACE inhibitors and safety of angiotensin receptor blocker therapy. Congest Heart Fail.

[7] Agostoni A, Cicardi M (2001). Drug-induced angioedema without urticaria. Drug Saf.

[8] Ishoo E, Shah UK, Grillone GA, Stram JR, Fuleihan NS (1999). Predicting airway risk in angioedema: staging system based on presentation. Otolaryngol Head Neck Surg.

[9] Agah R, Bandi V, Guntupalli KK (1997). Angioedema: the role of ACE inhibitors and factors associated with poor clinical outcome. Intensive Care Med.

[10] Cupido C, Rayner B (2007). Life-threatening angio-oedema and death associated with the ACE inhibitor enalapril. SAfrMedJ.

[11] Moellman JJ, Bernstein JA, Lindsell C, Banerji A, Busse PJ, Camargo CA (2014). A Consensus Parameter for the Evaluation and Management of Angioedema in the Emergency Department. Acad Emerg Med.

[12] Brown NJ, Ray WA, Snowden M, Griffin MR (1996). Black Americans have an increased rate of angiotensin converting enzyme inhibitor-associated angioedema. Clin Pharmacol Ther.

[13] Vasekar M, Craig TJ (2012). ACE inhibitor-induced angioedema. Curr Allergy Asthma Rep.

[14] Banerji A, Clark S, Blanda M, LoVecchio F, Snyder B, Camargo CA (2008). Multicenter study of patients with angiotensin-converting enzyme inhibitor-induced angioedema who present to the emergency department. Ann Allergy Asthma Immunol.

[15] Nussberger J, Cugno M, Amstutz C, Cicardi M, Pellacani A, Agostoni A (1998). Plasma bradykinin in angio-oedema. Lancet.

[16] Nussberger J, Cugno M, Cicardi M (2002). Bradykinin-mediated angioedema. N Engl J Med.

[17] Bas M, Greve J, Stelter K, Bier H, Stark T, Hoffmann TK (2010). Therapeutic efficacy of icatibant in angioedema induced by angiotensin-converting enzyme inhibitors: a case series. Ann Emerg Med.

[18] Roberts JR, Lee JJ, Marthers DA (2012). Angiotensin-converting enzyme (ACE) inhibitor angioedema: the silent epidemic. Am J Cardiol.

[19] Perez DV, Infante S, Marco G, Zubeldia JM (2011). Angioedema induced by angiotensin-converting enzyme inhibitors: two cases of successful traetment with a novel B2 bradykinin antagonist. J Allergy Clin Immunol.

[20] Reksten A (2011). Treatment of Angiotensin-Converting Enzyme Inhibitor-Indced Oropharyngeal Angioedema with Icatibant.

[21] Schmidt PW, Hirschl MM, Trautinger F (2010). Treatment of angiotensin-converting enzyme inhibitor-related angioedema with the bradykinin B2 receptor antagonist icatibant. J Am Acad Dermatol.

[22] Bas M, Greve J, Stelter K (2015). A randomized trial of icatibant in ACE-inhibitor-induced angioedema. N Engl J Med.

[23] Willis GB. Cognitive interviewing: A tool for improving questionnaire design. CA: Sage Publications; 2004.

[24] Ericsson KA, Simon HA (1980). Verbal Reports as Data. Psychol Rev.

[25] Joffe H, Yardley L, Marks DF, Yardley L (2004). Content and Thematic Analysis. Research Methods for Clinical and Health Psychology.

[26] Leeuwen T, Jewitt C (2001). The Handbook of Visual Analysis.

[27] Shrout PE, Fleiss JL (1979). Intraclass correlations: uses in assessing rater reliability. Psychol Bull.

[28] IBM Corp. IBM SPSS Statistics for Windows, Version 19.0. Armonk, NY: IBM Corp. 2010.

[29] Nunnally JC, Bernstein I (1994). The assessment of reliability. Psychometric Theory.

[30] Terwee CB, Bot SD, de Boer MR, van der Windt DA, Knol DL, Dekker J (2007). Quality criteria were proposed for measurement properties of health status questionnaires. J Clin Epidemiol.

